# Effect of Sonication Batch on Electrical Properties of Graphitic-Based PVDF-HFP Strain Sensors for Use in Health Monitoring

**DOI:** 10.3390/s24062007

**Published:** 2024-03-21

**Authors:** Victor Díaz-Mena, Xoan F. Sánchez-Romate, María Sánchez, Alejandro Ureña

**Affiliations:** 1 Materials Science and Engineering Area, Escuela Superior de Ciencias Experimentales y Tecnología, Universidad Rey Juan Carlos, Calle Tulipán s/n, 28933 Móstoles, Spain; maria.sanchez@urjc.es (M.S.); alejandro.urena@urjc.es (A.U.); 2 Instituto de Tecnologías para la Sostenibilidad, Universidad Rey Juan Carlos, 28933 Móstoles, Spain

**Keywords:** PVDF-HFP, strain sensors, health monitoring, carbon nanotubes, graphene nanoplatelets, electrical properties

## Abstract

In this study, flexible nanocomposites made from PVDF-HFP reinforced with carbon nanotubes (CNTs) and graphene nanoplatelets (GNPs) are manufactured using a sonication and solvent casting method for monitoring purposes. More specifically, the effect of the volume batch under the sonication process is explored. For CNT-based composites, the electrical conductivity decreases as the batch volume increases due to less effective dispersion of the CNTs during the 30-min sonication. The maximum electrical conductivity achieved in this type of sensor is 1.44 ± 0.17 S/m. For the GNP-based nanocomposites, the lower the batch volume is, the more breakage of nanoplatelets is induced by sonication, and the electrical response decreases. This is also validated by AC analysis, where the characteristic frequencies are extracted. Here, the maximum electrical conductivity measured is 8.66 ± 1.76 S/m. The electromechanical results also show dependency on the batch volume. In the CNT-based nanocomposites, the higher gauge factor achieved corresponds to the batch size, where the sonication may be more effective because it leads to a dispersed pathway formed by aggregates connected by tunneling mechanisms. In contrast, in the CNT-based nanocomposites, the GF depends on the lateral size of the GNPs. The biggest GF of all sensors is achieved with the PVDF-HFP/GNP sensors, having a value of 69.36 × 10^4^ at 35% of strain, while the highest GF achieved with a PVDF-HFP/CNT sensor is 79.70 × 10^3^ at 70%. In addition, cycling tests show robust electromechanical response with cycling for two different strain percentages for each type of nanocomposite. The sensor with the highest sensitivity is selected for monitoring two joint movements as proof of the applicability of the sensors manufactured.

## 1. Introduction

Wearable sensors are one of the main technologies that allow doctors to obtain real-time data from patients by detecting slight changes from baseline values over time. In recent years, there have been considerable advancements within this field due to the development of sensing materials and flexible electronics, which could pave the way for a new era of healthcare and medicine [1,2].

Wearable sensors based on conventional metals or semiconductors are becoming obsolete due to their limited sensitivity, low stretchability, and complex manufacturing processes. For these reasons, many researchers are studying polymer nanocomposite-based flexible strain sensors, which are usually classified according to the matrix material, electrodes used, and sensing unit or active sensing reinforcement [3]. This research article is focused on the development of piezoresistive strain sensors, whose electrical resistivity changes according to the subjected mechanical deformation, such as body movements [4], temperature [5], cardiac activity [6,7], and blood pressure [8].

The matrix for wearable piezoresistive sensors must be chosen carefully to provide flexibility, stretchability, and long-term reliability [9,10]. It must have the ability to deform in different ways, such as stretching, bending, or twisting, while showing a robust electromechanical variation, that is, by maintaining the electrical properties intact when releasing the applied mechanical force. In this regard, materials such as PDMS [11], PVDF [12,13], PET [14,15], TPU [16,17,18], or Ecoflex [19,20] have been studied by several researchers for sensing applications. More specifically, PVDF and its copolymers are excellent candidates for functional applications. To achieve piezoresistive properties, these flexible polymers are usually reinforced with conductive nanoparticles. Here, the electrical response changes due to variations in the electronic transport between nanoparticles above a critical volume fraction called the percolation threshold. This change can be detected with a measurement system, and the information can be transmitted to a PC or a mobile phone so it can be analyzed by the doctor or the patient, with complete traceability of the patient’s health [21].

The active materials most studied as nano-fillers for piezoresistive strain sensors have been carbon derivatives, including graphene [22,23], carbon nanotubes [24], carbon black [25,26], etc., and metallic nanoparticles [27]. Carbon nanotubes (CNTs) and graphene nanoplatelets (GNPs) have attracted much attention as conducting nanofillers, as the percolation threshold for typical CNT-filled or GNP-conducting nanocomposites is lower than others due to their geometry, especially because of their high aspect ratio [28], which for CNT nanocomposites is below 1.0 wt. %.

However, this type of material usually presents limitations during the manufacturing process. More specifically, the presence of aggregates or agglomerated bundles of nanoparticles may be a detriment to the electrical properties. In this regard, the dispersion procedure must ensure proper nanoparticle distribution inside the host polymer to promote the creation of effective electrical pathways. To achieve this purpose, ultrasonication has proved to be a highly effective way to achieve good nanoparticle dispersion, especially in low-viscosity media, due to the aggressive cavitation forces induced during the process [29,30,31,32]. However, the effect of aspects such as the ultrasonication power or the volume of the batch sonicated remains to be deeply studied.

Therefore, this research aims to study the effect of batch volume during ultrasonication in CNT- and GNP-based composites with PVDF-HFP as a matrix for wearable sensor applications. For this purpose, first, the sensor fabrication process by solution and sonication of CNTs and GNPs inside a PVDF-HFP solution will be described. Then, the study of the electrical properties of each system will be conducted by a DC and AC electrical conductivity analysis, while a microstructural analysis of the nanofillers will be carried out via SEM and TEM characterization. Finally, the electromechanical response of the sensors will be studied by strain sensing tests under tensile conditions. In this way, a complete overview of the influence of manufacturing conditions on the electromechanical properties will be achieved.

## 2. Materials and Methods

### 2.1. Tools and Materials

In this section, the different models and commercial names of the materials and test systems used for the manufacture and characterization of the nanocomposites are presented.

#### 2.1.1. Materials

The polymeric material used as the matrix for the nanocomposites was Poly(vinylidene fluoride-co-hexafluoropropylene) (PVDF-HFP) supplied by Byosynth^®^ (Berkshire, UK) in the form of pellets. To manufacture the nanocomposites via solvent casting, the solvent used for dissolving the PVDF-HFP pellets was N, N-Dimethylformamide (DMF) purchased from Sigma Aldrich^®^ (San Luis, MO, USA).

The two different nano reinforcements used were Multi-Wall Carbon Nanotubes (MWCNTs or CNTs) NC7000, supplied by Nanocyl^®^ (Sambreville, Belgium) with 95% purity, and Graphene Nanoplatelets (GNPs) purchased from XG-Science^®^ (East Lansing, MI, USA) M25. Both nano reinforcements were dispersed via ultrasonication enhanced using the surfactant Triton X-100 purchased from DOW^®^ (Midland, MI, USA).

#### 2.1.2. Test Devices

For the different steps in manufacture and characterization, several test devices were used. All of them are listed in this section.

- Manufacturing facilities: For the ultrasonication process, a HIELSCHER^®^ (Tellow, Germany) Ultrasonic Processor UP400ST machine was used. The evaporation process was carried out in a BINDER ED23 oven.
- Microstructural characterization: Scanning electron microscopy from PHILIPS^®^ (Amsterdam, Netherlands) (SEM—XL 30 ESEM) and transmission electron microscopy from JEOL^®^ (Tokyo, Japan) (TEM—JEM 1010) facilities from the university were used.
- Electrical characterization: DC tests were performed using a KEITHLEY^®^ (Cleveland, OH, USA) Source Meter Unit instrument (Series 2400 SourceMeter). The AC tests were performed by electrical impedance spectroscopy (EIS) using a METROHM (Madrid, Spain) Autolab potentiostat (PGSTAT302N station).
- Electromechanical tests: The quasi-static and cyclic tensile tests were performed on a ZWICK^®^ (Barcelona, Spain) universal tensile machine with a 500 N load cell (ProLine Z100). During these tests, the electrical response was recorded with an Agilent test unit at an acquisition frequency of 10 Hz (34410A multimeter).

### 2.2. Film Preparation

PVDF-HFP was dissolved in DMF to form a PVDF-HFP solution with a ratio of 1 g of PVDF-HFP:10 mL of DMF, generating an approximate 10 wt. % PVDF solution, which has been studied as one of the ratios near the maximum achievable with reasonable evaporation time [33,34,35,36,37]. During the dissolving process, the solution was continuously stirred using a magnetic stirrer for 2 h at 300 rpm at room temperature. After the dissolving process, CNTs and GNPs were added to the solution in different concentrations (Table 1) with Triton X-100, which was added in different ratios regarding the mass of CNT and GNP for each type of sensor.

The dispersion process of the two different nanocomposites in the PVDF-HFP solution was conducted by ultrasonication at 0.5 pulse cycles and 50% amplitude for 30 min. Here, the effect of volume batch during sonication was studied, using recipients of 10, 20, 30, and 40 mL.

After the ultrasonication process, the solution was deposited on a plane glass cleaned with acetone in an ultrasonic bath. Then, a thermal treatment was applied to evaporate the DMF solvent for 1 h at 90 °C. Nanocomposite films with dimensions 70 mm × 25 mm × 0.1 mm were extracted from the substrate after the solvent evaporation treatment.

### 2.3. Nanoparticle Characterization

After the ultrasonication process, the filtering of the particles, both CNTs and GNPs, was conducted using nylon membrane filters of 0.22 and 0.45 μm pore size to analyze the effect of the sonication process on the nanoparticles. To this end, SEM and TEM analyses were conducted. Measurements and distribution of the nano reinforcement dimensions were performed with the software ImageJ 1.54j.

### 2.4. Electrical Characterization

Volumetric electrical conductivity in direct current (DC) was determined by calculating the slope of the I-V characteristic curve within the range of 0–100 V (compliance = 0.02 A) for PVDF-HFP/CNT nanocomposites and 0–20 V (compliance = 1 A) for PVDF-HFP/GNP nanocomposites. Ten measurement points were extracted, and three samples were evaluated per condition. For the tests, two electrodes based on copper wires with silver paint were placed on the edges of each sample.

Electrical properties in alternating current (AC) were measured using electrical impedance spectroscopy (EIS). From these measurements, the real and imaginary parts of electrical impedance (Z* = Z′ + jZ″) were measured in the frequency range 0.1–10^5^ Hz at 0.3 V voltage amplitude. Analysis of the measurements by modeling with equivalent circuits was conducted to describe the different mechanisms of electronic transport inside the nanocomposite.

### 2.5. Electromechanical Characterization

Quasi-static and cyclic tensile tests were performed on a Zwick universal tensile machine with a 5 kN load cell. For the quasi-static tests, a crosshead speed of 10 mm/min for the PVDF-HFP/CNT samples was used, while 5 mm/min was selected for the GNP-based samples.

For the cyclic tests conducted to analyze the robustness of the sensors, the amplitude was set at 5 and 10% of strain (PVDF-HFP/CNT) and 1 and 2.5% of stain (PVDF-HFP/GNP) for 200 cycles due to their more brittle nature.

Simultaneously to the mechanical tests, the electrical response was recorded by measuring the electrical resistance between two electrodes. In this sense, the gauge factor (GF), which defines the sensitivity of the sensors, was calculated for each condition following the equation:

(1) $$GF=\frac{∆R/R_{0}}{\varepsilon }$$

where $∆R/R_{0}$ is the change of the normalized electrical resistance, recorded by the Agilent hardware, and ε, the applied mechanical strain.

**Figure 1:**
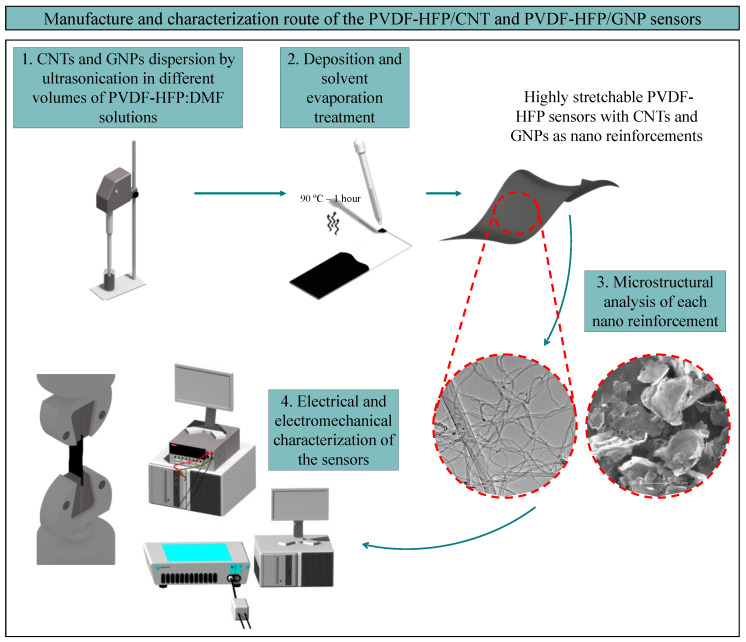
Manufacture and characterization routes for the two types of sensors manufactured. Steps 1 and 2 show the steps followed in manufacturing the thin and highly stretchable nanocomposite films and steps 3 and 4 show the microstructural and electromechanical characterization of the sensors.

Figure 1 shows a visual scheme of the complete route of manufacturing and characterization of the different types of sensors manufactured during this study.

### 2.6. Proof-of-Concept Tests

As a last step in characterization, after the study of the effect of different batch volumes sonicated in the electrical and electromechanical properties of PVDF-HFP sensors with CNTs and GNPs, two proof-of-concept tests were conducted. The condition with the highest gauge factor achieved was selected to carry out these tests based on monitoring wrist and finger bending. The electrical response of the sensor was recorded with the Agilent test unit. The main author of the research paper gave his full consent to the development of these tests on the above-mentioned two parts of his body.

## 3. Results and Discussion

In this section, first, a microstructural characterization of the nanoparticles is conducted after the dispersion process, followed by an analysis of DC and AC electrical properties. Finally, the electromechanical properties of the nanocomposites in terms of electrical sensitivity and robustness in cyclic response are explored and correlated with the previous characterizations carried out.

### 3.1. Microstructural Analysis

**Figure 2:**
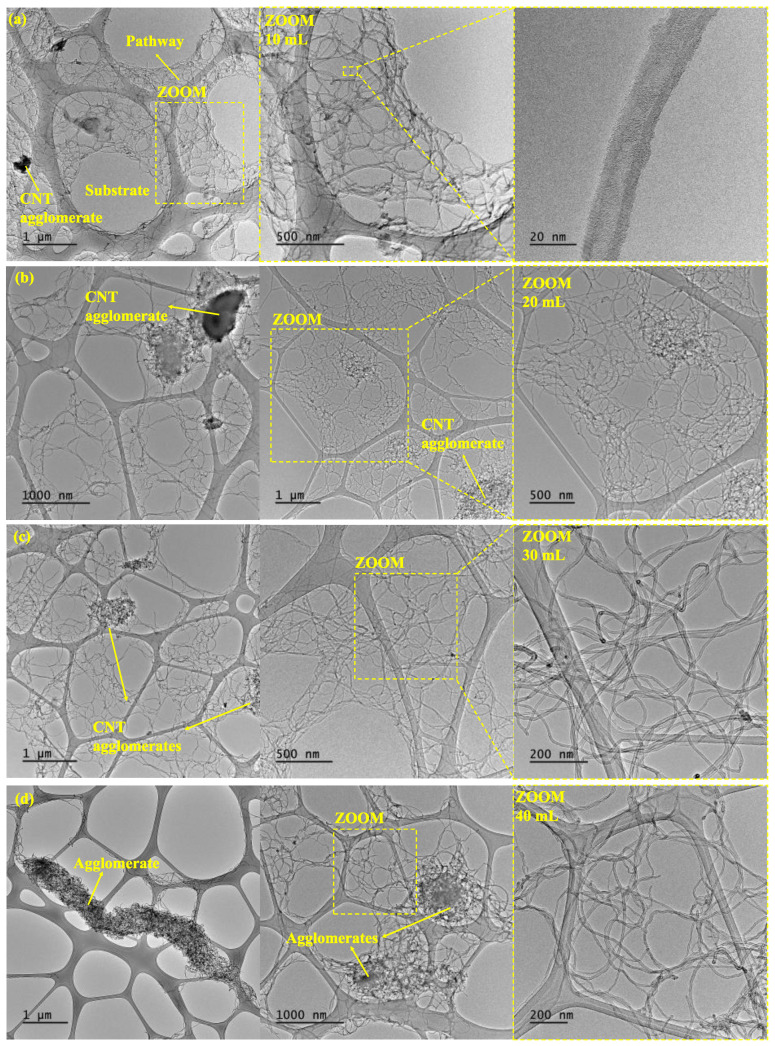
TEM images of the CNTs after 30 min of sonication in (**a**) 10 mL, (**b**) 20 mL, (**c**) 30 mL, and (**d**) 40 mL batch volumes of PVDF-HFP dissolved in DMF.

**Figure 3:**
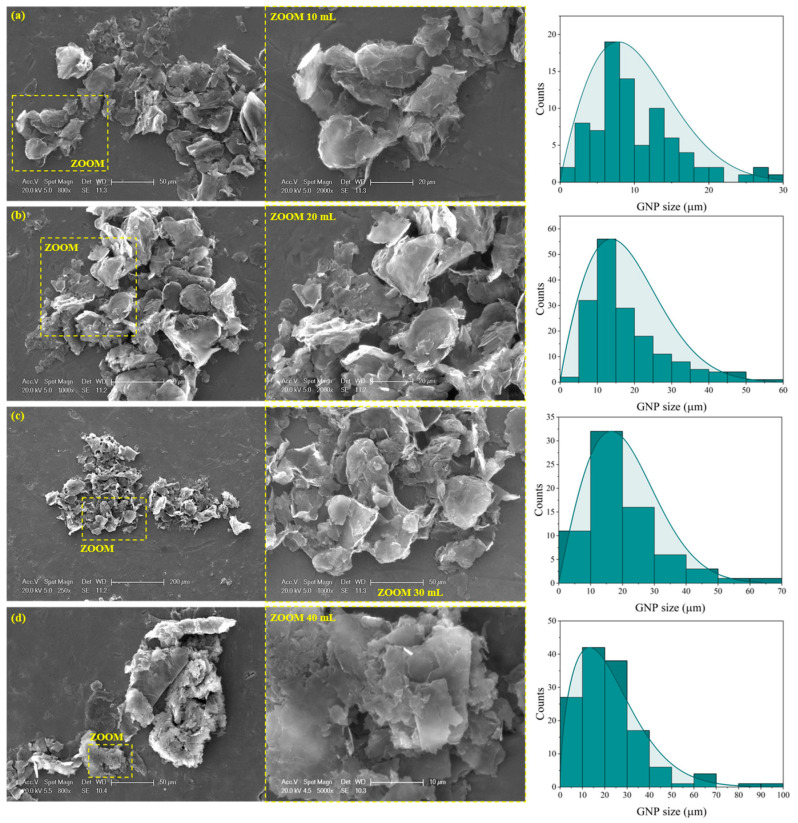
SEM images of the GNPs after 30 min of sonication in (**a**) 10 mL, (**b**) 20 mL, (**c**) 30 mL, and (**d**) 40 mL batch volumes of PVDF-HFP dissolved in DMF.

Figure 2 and Figure 3 show the microstructural analysis for both the CNT- and GNP-based nanocomposites with TEM and SEM images, respectively. Both analyses will be discussed in terms of clusters and damage induced on each nano reinforcement depending on the volume of the batch sonicated.

Figure 2 shows TEM images of the CNT batches. As the batch volume increases, a higher aggregation of CNTs is presented. More specifically, the 10 mL samples show a well-distributed CNT network (Figure 2a), while the 40 mL batch presents CNT clusters (Figure 2c). This can be explained according to the effectiveness of the ultrasonication technique, where the sonication energy, E (J/mL), delivered to the nanoparticles can be estimated by the following formula [38]:

(2) $$E=P\cdot \frac{t}{V}$$

where P is the sonicator output power (W), t is the sonication time (s), and V is the solution volume (mL) to be sonicated. For equal time and power of sonication, higher volumes make sonication less effective as less energy reaches the nano reinforcement, leading to the presence of more prevalent CNT reaggregation, as confirmed by TEM analysis.

Figure 3 shows SEM images of the GNP batches. Here, a reduction in the GNP dimensions occurs as the batch volume decreases since the sonication is more effective, and more damage is induced on the nano reinforcements. It is possible to measure the lateral size of the nanoparticles due to their geometry, and a Weibull distribution is found when analyzing the measurements taken [39]. A decrease in the lateral size of the GNP as the batch volume decreases is observed, going from a mean value of 7 µm for the 10 mL condition to a mean value of 15 µm for the 40 mL condition. This has been previously reported by other researchers with sonication time instead of volume of the batch, but similar results can be found where more severe sonication can lead to the fracture or exfoliation of the GNPs [40,41,42,43].

### 3.2. DC Electrical Properties

**Figure 4:**
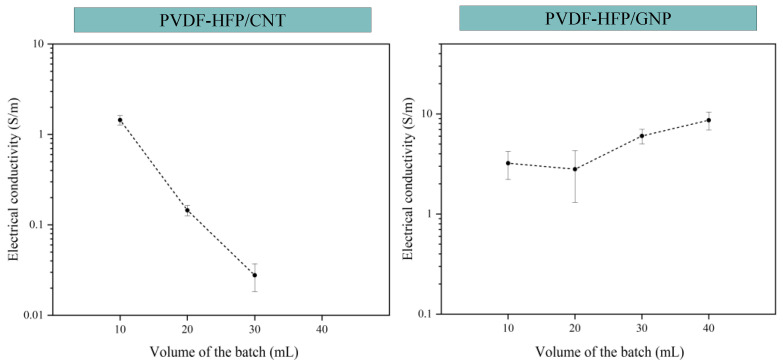
Electrical conductivities of CNT-based sensors (**left**) and GNP-based sensors (**right**) as a function of ultrasonication batch volumes.

Figure 4 shows the variation in the DC electrical conductivity as a function of the volume of the batch sonicated for both the CNT- and GNP-based nanocomposites. Two different behaviors are observed when comparing the CNT-based and GNP-based nanocomposites.

First, it is observed that the electrical conductivity of the CNT-based nanocomposites decreases with the increase in the batch volume, where a 40 mL batch volume does not possess electrical conductivity. This may be related to the effectiveness of the sonication process, which decreases as the batch volume increases, as commented before. This leads to a more entangled network at higher volumes, which promotes a reduction in electrical conductivity since the electrical pathway generated is not efficient.

In the case of GNP-based nanocomposites, the trend is different from the one observed with CNTs. Here, a similar value of electrical conductivity or even a significant increase is observed when increasing the batch volume. The ultrasonication process is a highly effective way to promote a widespread exfoliation of the GNPs [42], but it can also lead to very significant breakage of the nanoplatelets, especially in low-viscosity media [44,45,46]. This phenomenon has been previously confirmed with the SEM images of the GNPs and the lateral size measured for each volume of the batch. The reduction in the lateral size as the volume decreases would lead to a higher percolation threshold, and, thus, to a lower electrical conductivity. At higher volume batches, the exfoliation mechanisms would be more prevalent than the nanoparticle breakage, so the electrical network created is much more efficient, and the electrical conductivity increases.

For the GNP-based nanocomposites, the deviation from the average value of electrical conductivity for the GNP/10 and GNP/20 samples is higher than for the rest of the conditions. This can be due to the heterogeneity obtained due to the sonication at small batches, where it was commented that it would promote breakage of the nanoparticles. In this regard, it is necessary to analyze the sedimentation of the nanoparticles, which can be estimated from the sedimentation half-time, t_s_, defined as follows for nanoparticles suspension with Triton X-100 as surfactant [47]:

(3) $$t_{s}=9\eta L/d^{2}∆pg$$

where η is the viscosity of the suspension, L is the initial height of the sample suspension, d is the particle diameter, and Δp is the density difference between the particle and the suspension medium. Therefore, a higher GNP lateral size jointly with a low viscosity media would promote a reduction in the sedimentation time and, thus, higher sedimentation effects. In this case, the 20 mL sample presents an average lateral size in comparison to the rest of the samples, and the presence of fewer aggregates when compared to 30 and 40 mL would lead to a reduction in the viscosity.

### 3.3. AC Electrical Properties: Modeling of the Electrical Behavior via EIS

**Figure 5:**
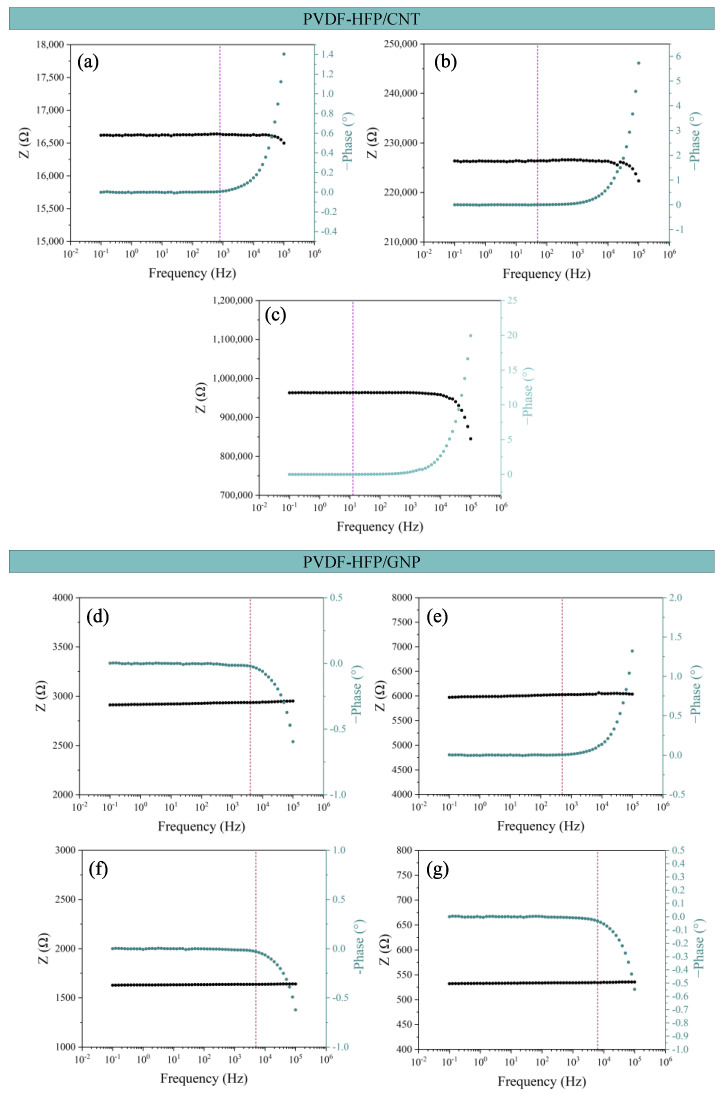
Bode plots for all conditions manufactured: (**a**–**c**) the CNT-based composites and (**d**–**g**) the GNP-based composites.

Figure 5 shows the Bode plots for all the sensors manufactured. Both CNT- and GNP-based nanocomposites show two different behaviors: (i) a frequency-independent part, where the electric current flows through the nanoparticle connections by both contact and tunneling transport mechanisms, and (ii) a frequency-dependent part of the impedance Z when the electrical behavior is more dominated by the matrix interface.

At low frequencies, the more prevalent mechanisms are found by contact and tunneling effects between adjacent nanoparticles. Once a certain frequency is reached, called the characteristic frequency, the current starts to flow through the polymeric matrix in those zones where nanoparticles are further away, and a frequency-dependent behavior can be observed [48,49,50,51].

**Figure 6:**
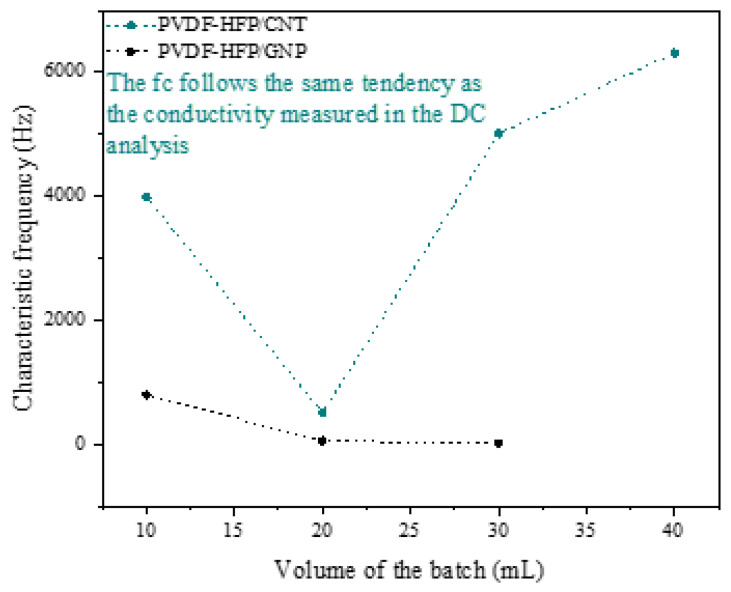
Changes in the characteristic frequency (fc) as a function of the volume of the batch.

In all the Bode diagrams, the vertical dashed line marks the characteristic frequency of that sample, where the phase starts changing from an almost 0 value. The analysis of this variable can be found in Figure 6. Here, it can be observed that the characteristic frequency value is proportional to the electrical conductivity, where it is higher in the case of the most conductive conditions as they present a more frequency-independent behavior.

**Figure 7:**
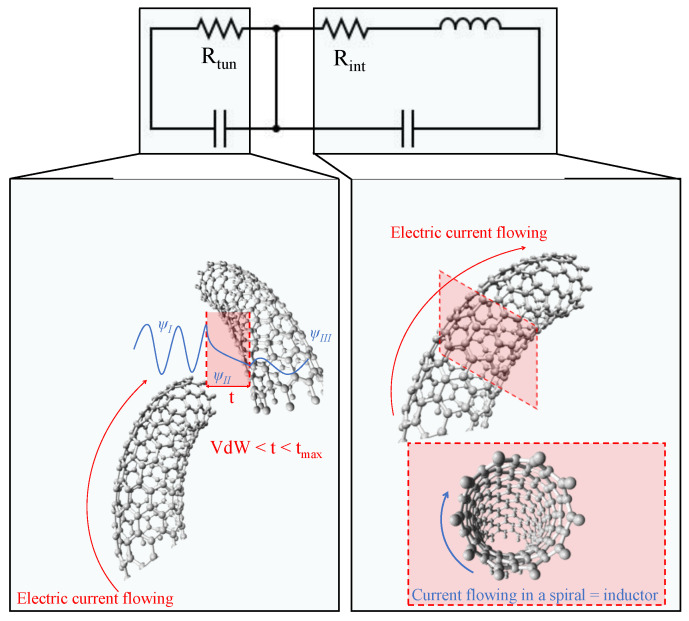
Explanation of the distinct parts of the electrical circuit used for modeling the AC behavior of the samples.

For a better comprehension of the electrical transport inside the material and the heterogeneity found in the DC analysis, a deeper analysis was conducted using equivalent circuits to model the measured AC behavior. The circuit employed has been previously studied by several researchers [52], and it consists of an RC in series with an RLC, as shown in Figure 7 for CNTs as conductive nanoparticles.

The RC part models the electrical transport between neighboring nanoparticles, known as tunneling transport, when the distance between them is higher than the de Van der Waals distance (0.34 nm for carbonaceous fillers) but lower than the maximum distance for tunneling (t_max_). The resistance is related to the probability of tunneling, while the capacitor simulates all the energy that is stored by the polarization of the polymer.

The RCL part models the behavior of a single particle or an accumulation of particles when these are at a distance equal to the VdW distance (agglomerates), where the resistance is the intrinsic resistance of the CNT or GNP. The capacitance and inductance elements show the dissipation of energy, whether in the form of accumulation or in terms of a magnetic field generated when the electrons flow following the CNT cylindric surface.

**Figure 8:**
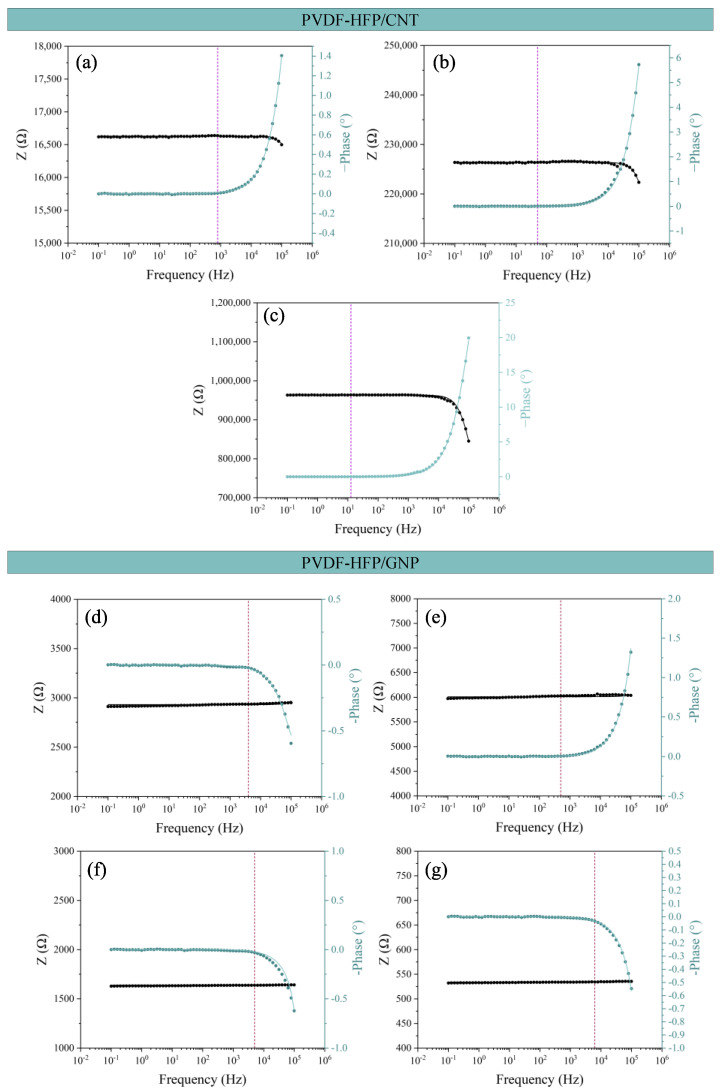
Bode plots for all conditions manufactured: (**a**–**c**) the CNT-based composites and (**d**–**g**) the GNP-based composites with electrical circuit modeling represented as a line in all graphs.

**Figure 9:**
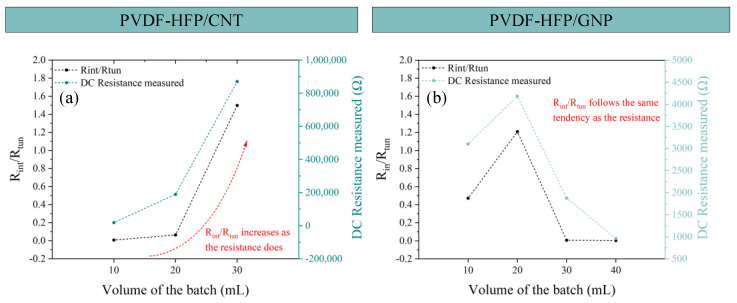
R_tun_ and R_int_ (**top**) and R_int_/R_tun_ (**bottom**) analysis for CNT-based sensors (**a**) and GNP-based sensors (**b**).

In this regard, Figure 8 shows the theoretical fitting of the Bode plots commented on before in Figure 7. A good agreement between the theoretical fitting (line) and the measurements (dots) is achieved. In Figure 9, the R_tun_ and R_int_ values (in terms of R_int_/R_tun_) of this analysis are shown with the DC electrical resistance measured so the tendencies could be compared. A high R_int_/R_tun_ would denote an electrical network more dominated by contact and intrinsic mechanisms, that is, more aggregated. As the R_int_/R_tun_ decreases, an electrical pathway based on different contact and tunneling effects is presented.

For PVDF-HFP/CNT nanocomposites (Figure 9a), as the volume of the batch increases, the value of the two resistances also increases. For the CNT/30 sample, the value of the R_int_ is higher than the R_tun_, demonstrating that for that volume, the ultrasonication is not effective enough to promote the agglomerate breakage and CNT dispersion, so lower conductivity values are reached. This can be confirmed by the comparison of the R_int_/R_tun_ ratio with the electrical resistance measured in the DC analysis, where both parameters follow the same tendency.

For PVDF-HFP/GNP nanocomposites (Figure 9b), the variation of R_tun_ is also in good agreement with the DC conductivity values, where a higher prevalence of intrinsic and contact mechanisms is found for the 20 mL sample, where a less efficient electrical network is observed. Again, the tendency observed for the results obtained by the electrical circuit analysis can be compared with the electrical resistance measured in the DC analysis.

### 3.4. Quasi-Static and Cyclic Tests

**Figure 10:**
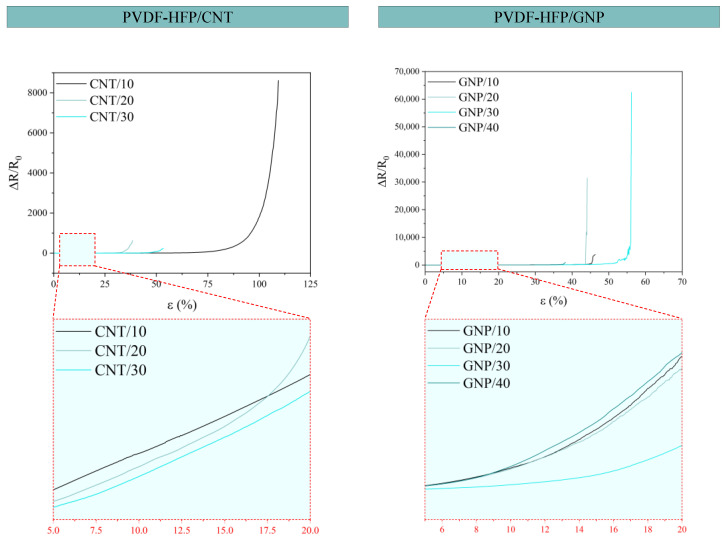
Tensile stress curves with electrical response (ΔR/R_0_) for both CNT-based sensors (**left**) and GNP-based sensors (**right**).

Sensors with approximate dimensions of 70 mm × 25 mm × 0.1 mm, as stated in the film preparation section, were tested under tensile stress tests. Figure 10 shows the electrical response, in terms of ΔR/R_0_, during the quasi-static tests. The PVDF-HFP/CNT nanocomposites have greater elongation at break, reaching values up to 100%, while those of GNP are more fragile and do not reach values greater than 60% of elongation. In both cases, it can be observed that the ΔR/R_0_ grows linearly up to a certain value of deformation, and then the growth becomes exponential until the loss of electrical signal in the Agilent measuring equipment. The sensitivity of the sensors is estimated by calculating the Gauge Factor (GF).

**Figure 11:**
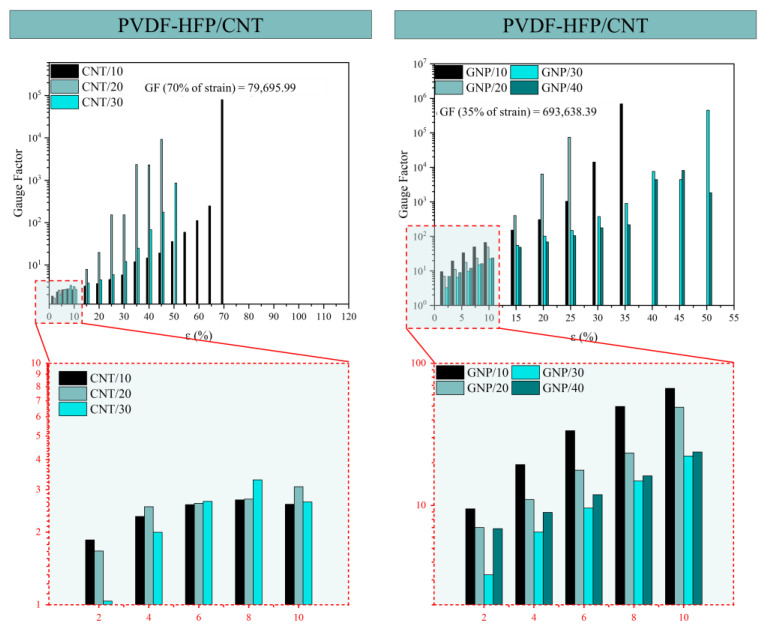
Gauge factors (GF) for CNT-based sensors (**left**) and GNP-based sensors (**right**) with the highest GF values marked for each, and a detail of the GF achieves at low percentages of strain.

Figure 11 summarizes the GF values as a function of the strain level. The biggest GF of all sensors was achieved with the PVDF-HFP/GNP sensors, having a value of 69.36 × 10^4^ at 35% of strain (GNP/20), while the highest GF achieved with a PVDF-HFP/CNT sensor was 79.70 × 10^3^ at 70% of strain (CNT/10). The higher sensitivity in the GNP sensors is explained through the 2D nature of the nanoplatelets. It is reflected in a higher tunneling area and, thus, a higher allowable separation between neighboring nanoparticles, leading to a more prevalent exponential effect of the tunneling resistance.

The GF analysis at low strain percentages (2, 4, 6, 8, and 10%) is shown as a zoom of the complete quasi-static plot in Figure 11. These percentages of strain are where the main conclusions can be extracted because these are the percentages more likely to occur when monitoring a body motion or vital sign. For the PVDF-HFP/CNT sensors, all the samples present similar GF up to 10% of the deformation range. For the GNP-based nanocomposites, the GNP/10 stands out as the most promising because of its higher GF value at all strain values within the 2–10% range. The previously commented reduction in the lateral size and higher exfoliation of the individual GNPs would promote the creation of an electrical network with a higher prevalence of tunneling mechanisms. Thus, the sensitivity is higher due to the more pronounced exponential effects.

**Figure 12:**
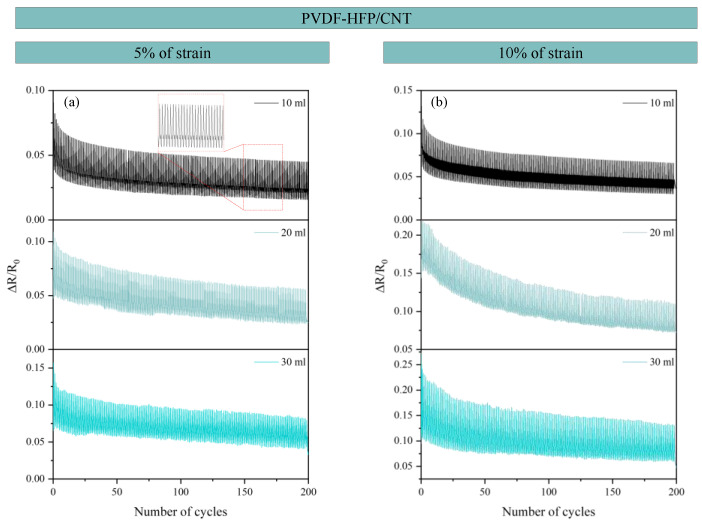
Cyclic tests for CNT-based sensors for both (**a**) 5% and (**b**) 10% of strain reached.

**Figure 13:**
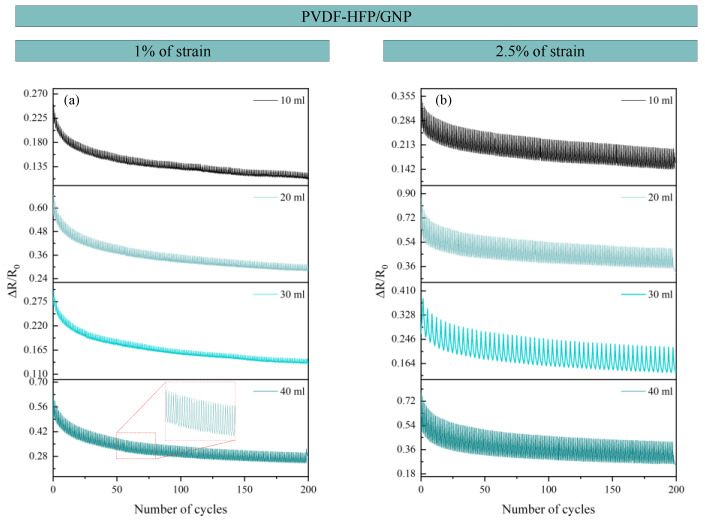
Cyclic tests for GNP-based sensors for both (**a**) 1% and (**b**) 2.5% of strain reached.

Figure 12 and Figure 13 show the cyclic tests conducted in both types of sensors, CNT- and GNP-based, respectively. For the PVDF-HFP/CNT sensors, 5% and 10% of strain were reached, while the PVDF-HFP/GNP sensors were evaluated up to 1% and 2.5% of strain due to their lower elongation at break. Both types of cyclic tests showed great repeatability of the electrical signal, with no significant changes in the electrical response between consecutive cycles. A slight decrease in the baseline values was observed throughout the cyclic test. This is explained by the inherent viscoelastic behavior of the matrix and the presence of possible irreversible effects in the electrical network, which are more pronounced in the first stages [18,53]. The electrical pathway formed by the nanoparticles is unstable and needs to settle down after the first cycles to form a more stable path. Regardless of this, the robustness of the sensors is proven, demonstrating the applicability of the proposed materials for sensing purposes.

### 3.5. Proof-of-Concept Tests

**Figure 14:**
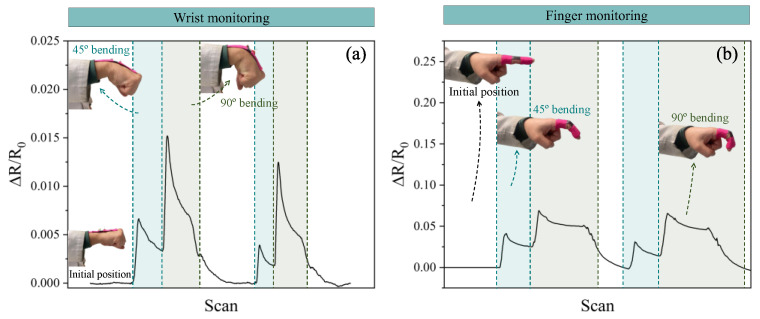
Wrist (**a**) and finger (**b**) motion monitoring at different degrees to prove the sensor’s applicability in health monitoring.

Figure 14 summarizes the trials performed to prove the human body monitoring of the developed sensors. For these tests, the GNP/10 condition was selected as it showed a higher GF at a lower percent of strain among all the sensors manufactured.

More specifically, wrist and single-finger movements were monitored. During both the wrist (Figure 14a) and finger movement (Figure 14b), an increase in ΔR/R_0_ was measured when increasing the bending angle. A continuous drop in the electrical resistance was recorded when the bending was maintained due to the viscoelastic response of the sensors. This was also observed, but in a less significant way, when monitoring the finger. This can be due to the more severe deformation under the wrist movement. Both bending monitorings were successfully achieved with a similar electrical response in consecutive repetitions, so their applicability for joint motion monitoring was proved.

## 4. Conclusions

The effect of volume batch during sonication on the electromechanical properties of PVDF-HFP copolymer reinforced with CNTs or GNPs has been studied.

For CNT-based nanocomposites, the DC electrical conductivity decreases with the volume of the batch sonicated. This is related to the lower effectiveness of the sonication process as the batch volume increases, achieving a worse dispersed electrical network within the material. For the GNP-based composites, small batch sizes promoted a pronounced breakage of the nanoparticles due to the excessively aggressive cavitation forces induced by the ultrasound technique, which would explain the lower conductivity values under these conditions. In high-volume batches, the exfoliation mechanisms would be more prevalent than nanoparticle breakage, so the sonication pulses would promote the increase in electrical pathways needed to obtain more conductivity.

AC measurements under EIS analysis showed that the sensors present a resistive-capacitive/inductive behavior for every condition. Moreover, it was observed that the characteristic frequency increases with electrical conductivity, which is reflected in a more dominant resistive behavior through the aggregates. For both GNP- and CNT-based nanocomposites, the electrical behavior under AC conditions was modeled via an RC-RLC electrical circuit, with good agreement with the experimental results.

The electromechanical response was also studied under quasi-static and cycling conditions. Regarding the quasi-static study, the maximum GF value was around 69.36 × 10^4^ at 35% of strain for the GNP/20 nanocomposite. Here, the highest GF values were obtained in those conditions where the participation of tunneling mechanisms was more prevalent, leading to a more pronounced exponential effect. Under cycling loading, the sensors showed a very robust electrical response, with a slight decay due to the intrinsic viscoelastic behavior of the material and noise in those conditions with lower electrical conductivity.

## Figures and Tables

**Table 1:** Summary of nomenclature for the different nanocomposites manufactured.

Sample Nomenclature	wt. % CNTs	wt. % GNPs	Nano-Reinforcement: Triton X-100 Ratio	Batch Volume (mL)
CNT/10	0.1	-	1:20	10
CNT/20	0.1			20
CNT/30	0.1			30
CNT/40	0.1			40
GNP/10	-	6	1:2	10
GNP/20		6		20
GNP/30		6		30
GNP/40		6		40

## Data Availability

The data presented in this study are available on request from the corresponding authors.
